# Data on optimisation of a multiplex HRM-qPCR assay for native and invasive crayfish as well as the crayfish plague in four river catchments

**DOI:** 10.1016/j.dib.2018.05.134

**Published:** 2018-05-29

**Authors:** Chloe Victoria Robinson, Tamsyn M. Uren Webster, Sofia Consuegra

**Affiliations:** Swansea University, Singleton Park, Swansea SA2 8PP, Wales, UK

**Keywords:** *Pacifastacus leniusculus*, *Austropotamobius pallipes*, Crayfish plague, HRM-qPCR, eDNA

## Abstract

The data presented here corresponds to the research paper “Simultaneous detection of invasive signal crayfish, endangered white-clawed crayfish and the crayfish plague using environmental DNA”. A crayfish-specific assay was designed and optimised using three real-time PCR supermixes (SYBR™ Green, SsoFast™ EvaGreen® and HOT FIREPol® EvaGreen®). Diagnostic high resolution melt (HRM) data from direct application of assay on both *ex-situ* eDNA water samples and field samples from four catchments (two in Wales, two in England) is presented in this article, displaying positive HRM profiles for invasive signal crayfish (*Pacifastacus leniusculus),* native white-clawed crayfish (*Austropotamobius pallipes*) and crayfish plague causal agent (*Aphanomyces astaci*).

**Specifications Table**

**Table:** 

Subject area	*Biology*
More specific subject area	*Detection of invasive, native crayfish and crayfish plague in environmental DNA water samples using HRM-qPCR analysis*
Type of data	*Sequence alignment, tables and figures*
How data was acquired	*Sequence alignment was achieved using GenBank and BioEdit (ver. 7.2.5)*
	*DNA concentrations determined using Qubit™ 4 Fluorometer (ThermoFisher Scientific, UK)*
	*qPCR data achieved using CFX96 Touch™ Real-Time PCR Detection System (C1000 Touch™ chassis, Bio-Rad, UK)*
Data format	*Raw*
Experimental factors	*DNA extracted from water and tissue samples using Qiagen DNeasy® Blood and Tissue extraction kit (QIAGEN, UK)*
Experimental features	*Assessment of presence/absence of signal crayfish, white-clawed crayfish and crayfish plague DNA in water samples from four river catchments*
Data source location	*Signal crayfish tank water samples from Cardiff University*
	*Native crayfish tank water samples from Cynrig Hatchery*
	*Water samples from the River Wye catchment (Builth Wells to Boughrood, UK)*
	*Water samples from the River Itchen catchment (Bishop׳s Sutton to Easton, UK)*
	*Water samples from the River Taff catchment (Nant-ddu to Treharris, UK)*
	*Water samples from the River Medway catchment (Tonbridge to Leybourne, UK)*
Data accessibility	*Data in full is provided with this article*
Related research article	*Robinson, C.V., Uren Webster, T.M., Cable, J., James, J., Consuegra, S. Simultaneous detection of invasive signal crayfish, endangered white-clawed crayfish and the crayfish plague using environmental DNA. Biological Conservation 222, 241–252.*[1]

**Value of the data**

• The data shows that melting curve differences between native and invasive crayfish can be used for management purposes by screening eDNA water samples.
• The protocol successfully amplifies invasive and native crayfish and can detect their infection status.
• The comparison of HRM-qPCR outputs using SYBR™ Green and SsoFast™ Evagreen® suggested that the second qPCR mastermix provided greater sensitivity and reproducibility.
• Temporal concentration measurements indicated that eDNA degraded 3 × in 48 h under controlled conditions.

## 1 Data

**Fig. 1:**
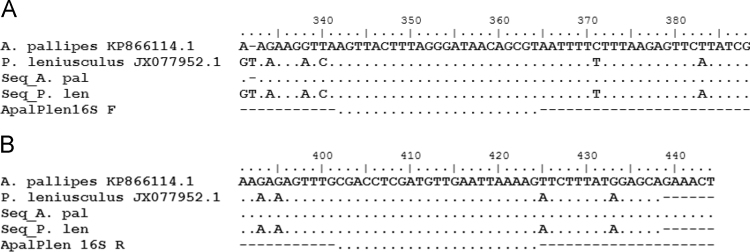
Alignment of DNA fragments from eDNA samples for both *Pacifastacus leniusculus* and *Austropotamobius pallipes* and ApalPlen16S forward (a) and reverse (b) primers against reference sequences. Seq_A.pal = positive *in-situ Austropotamobius pallipes* eDNA fragment; Seq_P. len = positive in-situ *Pacifastacus leniusculus* eDNA fragment.

Data presented in Section 1.1 includes a sequence alignment of *Pacifastacus leniusculus* and *Austropotamobius pallipes* 16s mtDNA 83 bp product with binding sites respective forward (ApalPlen16S_F) and reverse (ApalPlen16S_R) primers and nucleotide base differences between the two species (Fig. 1).

**Fig. 2:**
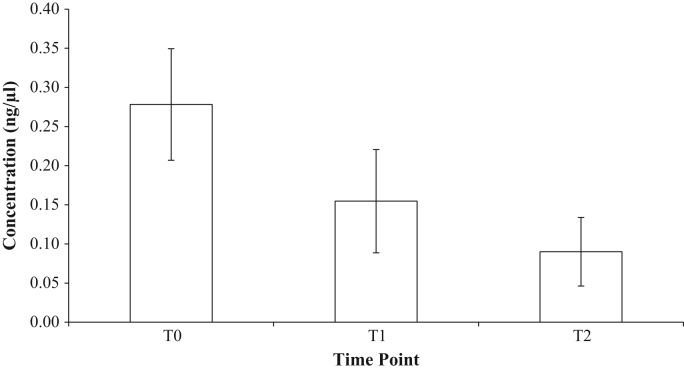
Average eDNA concentration across all *ex-situ* samples for the three time points (T0 = time of crayfish removal; T1 = 24 h post crayfish removal; T2 = 48 h post crayfish removal.

In Section 1.2, data is presented on the average eDNA concentrations of tank water samples collected from tanks containing *P. leniusculus* at three time points (Fig. 2).

**Fig. 3:**
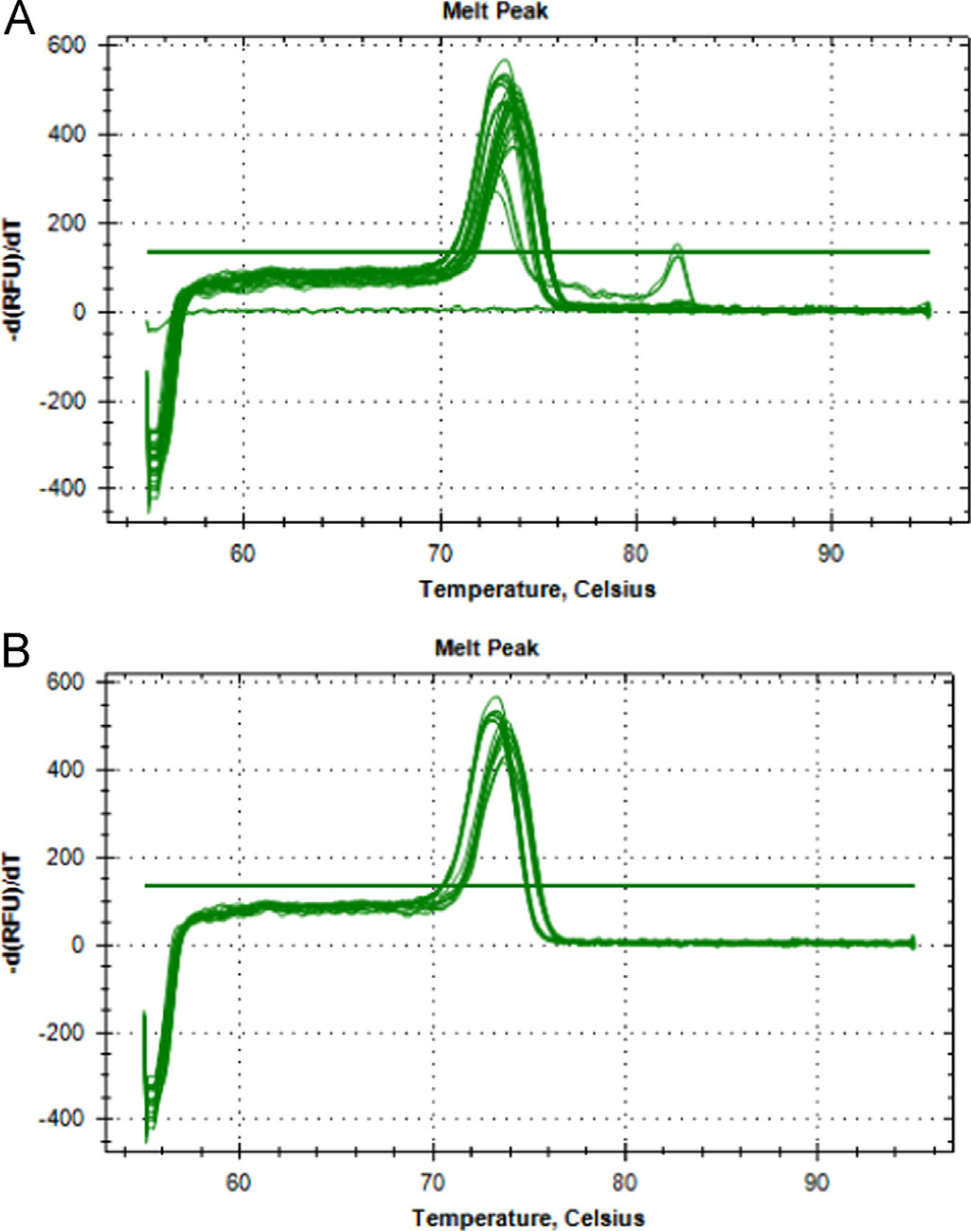
Melt peaks from SYBR™ Green dilution series (A) and from SsoFast™ EvaGreen® dilution series (B) for *Pacifastacus leniusculus* and *Austropotamobius pallipes* DNA pools.

**Fig. 4:**
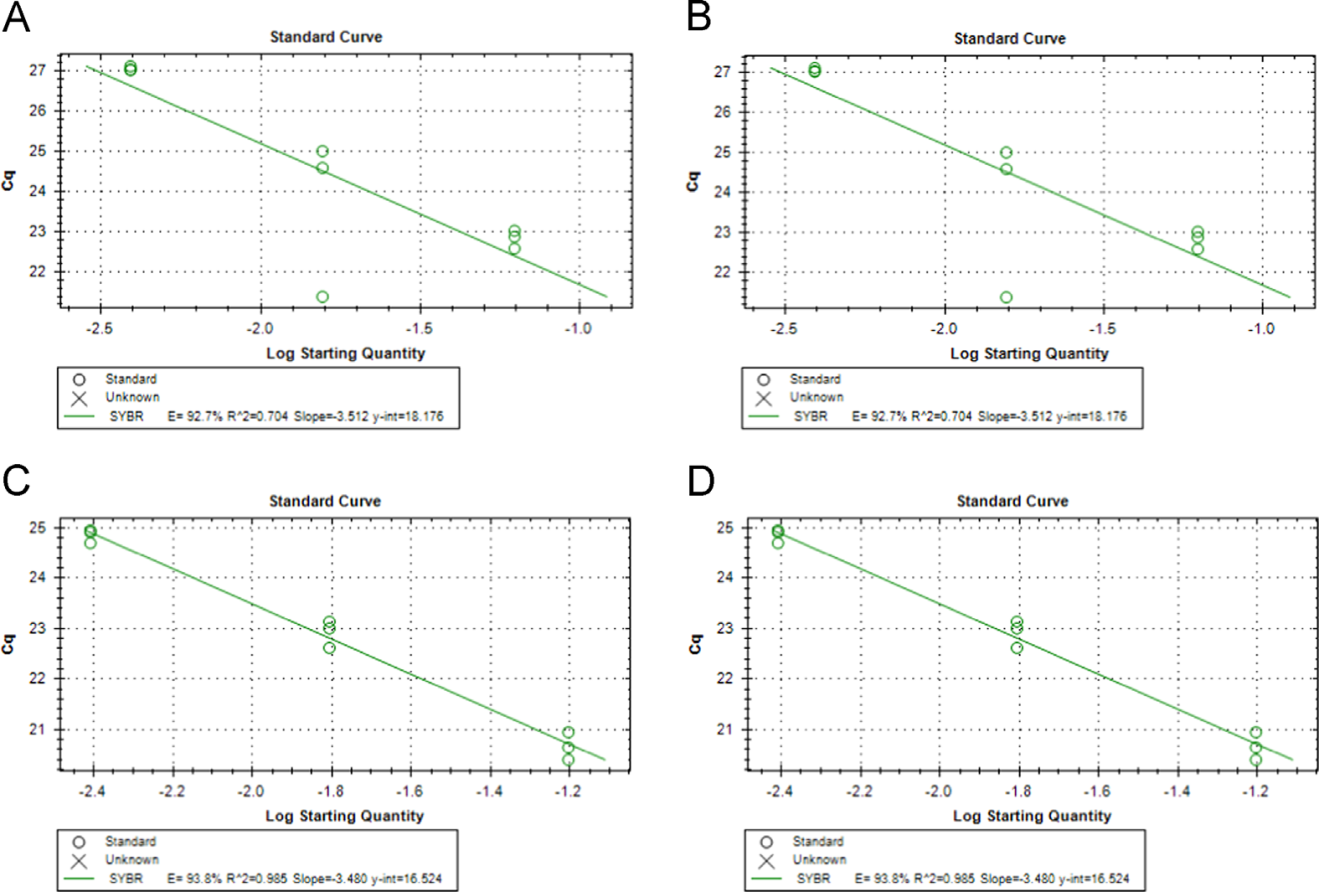
Efficiency outputs from SYBR™ Green dilution series for *Pacifastacus leniusculus* (A) and *Austropotamobius pallipes* (B); efficiency outputs from SsoFast™ EvaGreen® dilution series for (B) for *Pacifastacus leniusculus* (C) and *Austropotamobius pallipes* (D).

**Fig. 5:**
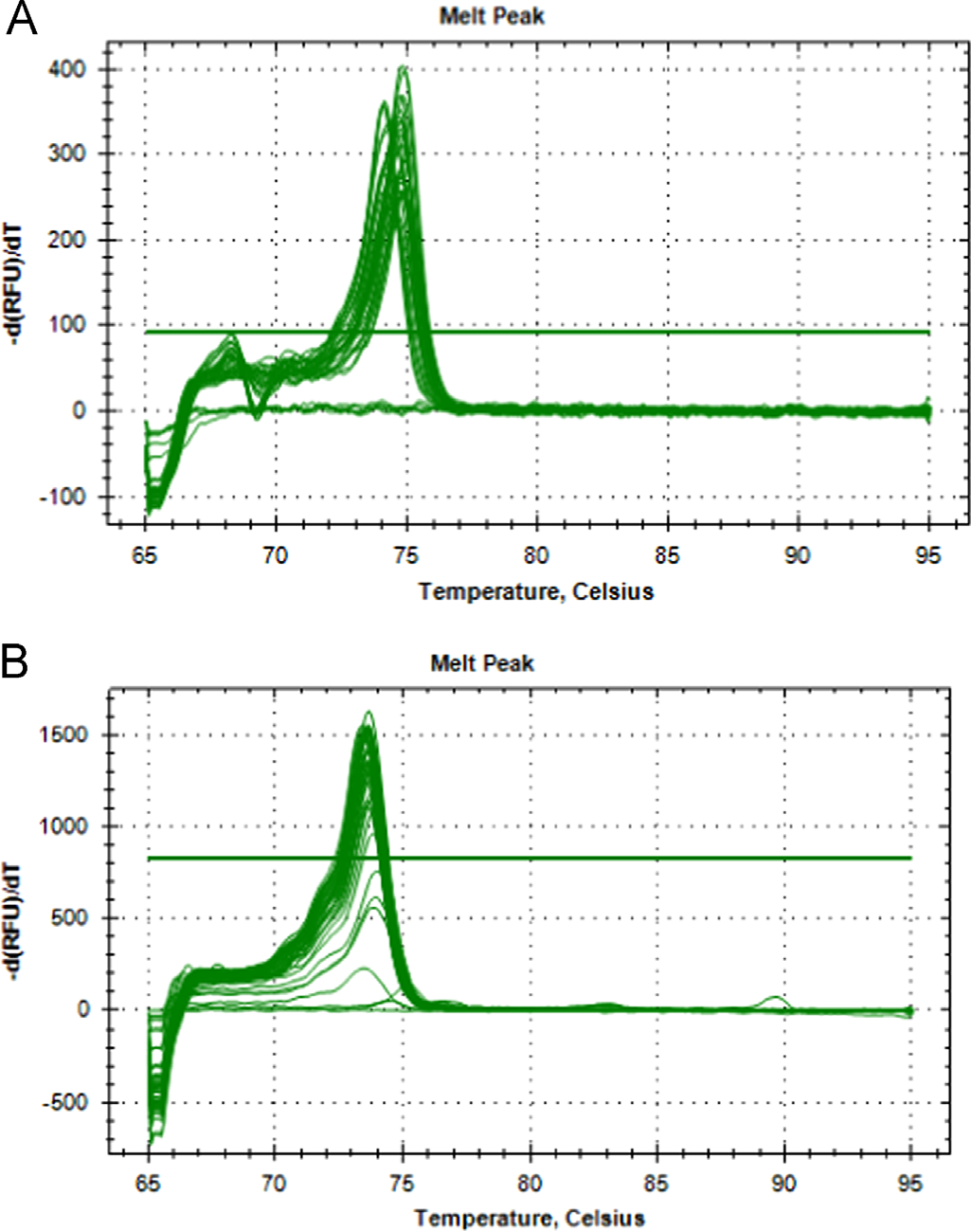
Melt peaks from SsoFast™ EvaGreen® mixed DNA ratios of crayfish species (*Pacifastacus leniusculus* and *Austropotamobius pallipes*) qPCR amplifications (A). 10:90 *Pacifastacus leniusculus*: *Austropotamobius pallipes* to 90:10 *Pacifastacus leniusculus*: *Austropotamobius pallipes*. Melt peaks from SsoFast™ EvaGreen® *ex-situ Pacifastacus leniusculus* eDNA qPCR amplifications (B).

**Fig. 6:**
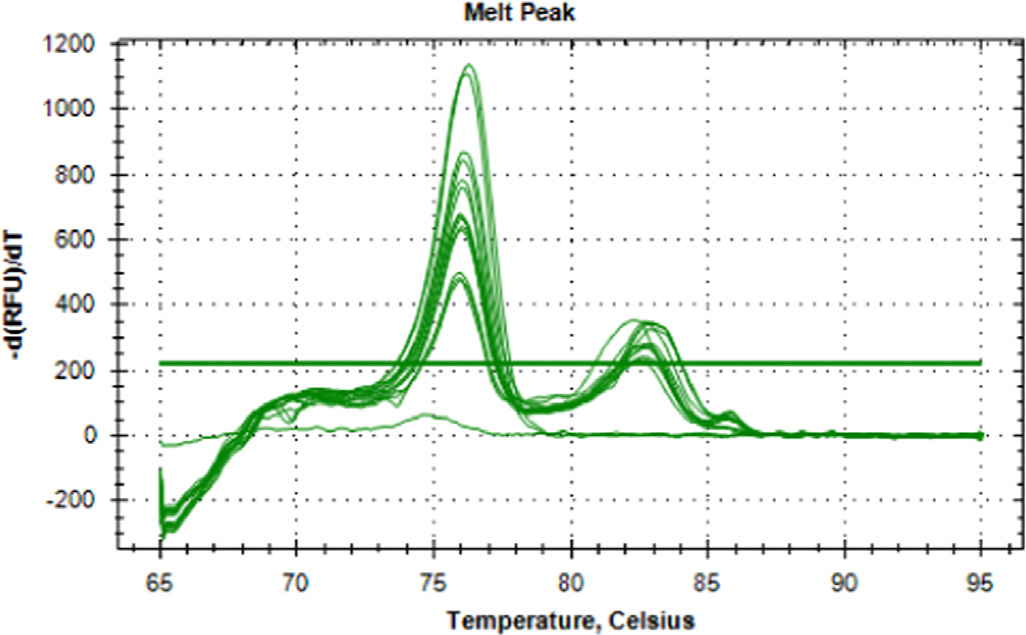
Melt peaks for HOT FIREPol^®^ EvaGreen^®^ qPCR multiplex optimised reactions using an *Aphanomyces astaci*-infected *Pacifastacus leniusculus* DNA pool.

The data presented in Section 1.3 consists of the SYBR™ Green Supermix and SsoFast™ EvaGreen® Supermix qPCR qPCR optimization results of both *P. leniusculus* and *A. pallipes* DNA, including the qPCR melt curve graphs (Fig. 3), standard curves with efficiency values (Fig. 4) and raw melt data (Table 1). In addition, Subsection 1.3 includes qPCR melt curve graphs (Fig. 5) and raw melt data (Table 2) for amplifications of mixed proportions of both *P. leniusculus* and *A. pallipes* DNA in the same reaction tube and *ex-situ P. leniusculus* tank eDNA amplifications (Fig. 5; Table 3). Data on the qPCR melt curve graphs and raw melt data for HOT FIREPol® EvaGreen® qPCR optimisation with *P. leniusculus* and the crayfish plague causal agent (*Aphanomyces astaci*) DNA are presented in Subsection 1.3 in Fig. 6 and Table 4.

**Table 1:** Melt peak data from SYBR™ Green and SsoFast™ EvaGreen® dilution series for *Pacifastacus leniusculus* (s_pool) and *Austropotamobius pallipes* (n_pool).

**Mastermix**	**Sample ID**	**Concentration (ng/µl)**	**Melt Temperature (°C)**
SYBR™ Green	s_pool	5	72.50
SYBR™ Green	s_pool	5	72.80
SYBR™ Green	s_pool	5	72.80
SYBR™ Green	n_pool	5	73.70
SYBR™ Green	n_pool	5	73.70
SYBR™ Green	n_pool	5	73.70
SYBR™ Green	s_pool	0.5	73.10
SYBR™ Green	s_pool	0.5	73.40
SYBR™ Green	s_pool	0.5	73.30
SYBR™ Green	n_pool	0.5	73.70
SYBR™ Green	n_pool	0.5	73.70
SYBR™ Green	n_pool	0.5	73.70
SYBR™ Green	s_pool	0.05	73.40
SYBR™ Green	s_pool	0.05	73.40
SYBR™ Green	s_pool	0.05	73.30
SYBR™ Green	n_pool	0.05	73.80
SYBR™ Green	n_pool	0.05	73.70
SYBR™ Green	n_pool	0.05	73.60
SYBR™ Green	s_pool	0.005	73.70
SYBR™ Green	s_pool	0.005	73.30
SYBR™ Green	s_pool	0.005	73.20
SYBR™ Green	n_pool	0.005	73.70
SYBR™ Green	n_pool	0.005	73.70
SYBR™ Green	n_pool	0.005	73.80
SYBR™ Green	s_pool	0.0005	72.90
SYBR™ Green	s_pool	0.0005	73.00
SYBR™ Green	s_pool	0.0005	73.00
SYBR™ Green	n_pool	0.0005	73.80
SYBR™ Green	n_pool	0.0005	73.70
SYBR™ Green	n_pool	0.0005	73.70
SYBR™ Green	MB	N/A	None
SYBR™ Green	MB	N/A	None
SYBR™ Green	MB	N/A	None
SsoFast™ EvaGreen®	s_pool	5	82.10
SsoFast™ EvaGreen®	s_pool	5	72.50
SsoFast™ EvaGreen®	s_pool	5	72.80
SsoFast™ EvaGreen®	s_pool	5	72.80
SsoFast™ EvaGreen®	n_pool	5	73.70
SsoFast™ EvaGreen®	n_pool	5	73.70
SsoFast™ EvaGreen®	n_pool	5	73.70
SsoFast™ EvaGreen®	s_pool	0.5	73.10
SsoFast™ EvaGreen®	s_pool	0.5	73.40
SsoFast™ EvaGreen®	s_pool	0.5	73.30
SsoFast™ EvaGreen®	n_pool	0.5	73.70
SsoFast™ EvaGreen®	n_pool	0.5	73.70
SsoFast™ EvaGreen®	n_pool	0.5	73.70
SsoFast™ EvaGreen®	s_pool	0.05	73.70
SsoFast™ EvaGreen®	s_pool	0.05	73.70
SsoFast™ EvaGreen®	s_pool	0.05	73.70
SsoFast™ EvaGreen®	n_pool	0.05	73.80
SsoFast™ EvaGreen®	n_pool	0.05	73.70
SsoFast™ EvaGreen®	n_pool	0.05	73.70
SsoFast™ EvaGreen®	s_pool	0.005	73.70
SsoFast™ EvaGreen®	s_pool	0.005	73.70
SsoFast™ EvaGreen®	s_pool	0.005	73.60
SsoFast™ EvaGreen®	n_pool	0.005	73.70
SsoFast™ EvaGreen®	n_pool	0.005	73.70
SsoFast™ EvaGreen®	n_pool	0.005	73.80
SsoFast™ EvaGreen®	s_pool	0.0005	72.90
SsoFast™ EvaGreen®	s_pool	0.0005	73.00
SsoFast™ EvaGreen®	s_pool	0.0005	73.00
SsoFast™ EvaGreen®	n_pool	0.0005	73.80
SsoFast™ EvaGreen®	n_pool	0.0005	73.70
SsoFast™ EvaGreen®	n_pool	0.0005	73.70
SsoFast™ EvaGreen®	MB	N/A	None
SsoFast™ EvaGreen®	MB	N/A	None
SsoFast™ EvaGreen®	MB	N/A	None
SsoFast™ EvaGreen®	MB	N/A	None
SsoFast™ EvaGreen®	MB	N/A	None
SsoFast™ EvaGreen®	MB	N/A	None

Sample ID: s_pool Signal crayfish DNA pool, n_pool Native crayfish DNA pool, MB Amplification negative controls.

**Table 2:** Melt curve data from SsoFast™ EvaGreen® mixed DNA ratios of crayfish species (*Pacifastacus leniusculus* and *Austropotamobius pallipes*) qPCR amplifications.

**Mastermix**	**Sample ID**	**Melt Temperature (°C)**
SsoFast™ EvaGreen®	10:90S:N	74.20
SsoFast™ EvaGreen®	10:90S:N	74.10
SsoFast™ EvaGreen®	10:90S:N	74.20
SsoFast™ EvaGreen®	90:10S:N	74.80
SsoFast™ EvaGreen®	90:10S:N	74.90
SsoFast™ EvaGreen®	90:10S:N	74.90
SsoFast™ EvaGreen®	20:80S:N	74.30
SsoFast™ EvaGreen®	20:80S:N	74.30
SsoFast™ EvaGreen®	20:80S:N	74.30
SsoFast™ EvaGreen®	30:70S:N	74.30
SsoFast™ EvaGreen®	30:70S:N	74.40
SsoFast™ EvaGreen®	30:70S:N	68.30
SsoFast™ EvaGreen®	30:70S:N	74.40
SsoFast™ EvaGreen®	40:60S:N	74.70
SsoFast™ EvaGreen®	40:60S:N	74.70
SsoFast™ EvaGreen®	40:60S:N	74.60
SsoFast™ EvaGreen®	50:50S:N	74.70
SsoFast™ EvaGreen®	50:50S:N	74.70
SsoFast™ EvaGreen®	50:50S:N	74.70
SsoFast™ EvaGreen®	60:40S:N	74.70
SsoFast™ EvaGreen®	60:40S:N	74.70
SsoFast™ EvaGreen®	60:40S:N	74.70
SsoFast™ EvaGreen®	70:30S:N	74.80
SsoFast™ EvaGreen®	70:30S:N	74.80
SsoFast™ EvaGreen®	70:30S:N	74.70
SsoFast™ EvaGreen®	80:20S:N	74.80
SsoFast™ EvaGreen®	80:20S:N	74.70
SsoFast™ EvaGreen®	80:20S:N	74.70
SsoFast™ EvaGreen®	PC_SC	74.00
SsoFast™ EvaGreen®	PC_SC	74.10
SsoFast™ EvaGreen®	PC_SC	74.10
SsoFast™ EvaGreen®	PC_NC	74.80
SsoFast™ EvaGreen®	PC_NC	74.80
SsoFast™ EvaGreen®	PC_NC	74.90
SsoFast™ EvaGreen®	MB	None
SsoFast™ EvaGreen®	MB	None
SsoFast™ EvaGreen®	MB	None
SsoFast™ EvaGreen®	MB	None
SsoFast™ EvaGreen®	MB	None
SsoFast™ EvaGreen®	MB	None

Sample ID: #:# Ratio of DNA mix, S Signal crayfish, N Native crayfish, PC_SC Signal crayfish positive DNA control, PC_NC Native crayfish positive DNA control, MB Amplification negative control.

**Table 3:** Melt peak data from SsoFast™ EvaGreen® *ex-situ Pacifastacus leniusculus* eDNA qPCR amplifications.

**Mastermix**	**Sample ID**	**Melt Temperature (°C)**
SsoFast™ EvaGreen®	1_T1	None
SsoFast™ EvaGreen®	1_T1	73.70
SsoFast™ EvaGreen®	1_T1	73.70
SsoFast™ EvaGreen®	7_T0	73.70
SsoFast™ EvaGreen®	7_T0	73.70
SsoFast™ EvaGreen®	7_T0	73.70
SsoFast™ EvaGreen®	1_T0	None
SsoFast™ EvaGreen®	1_T0	73.70
SsoFast™ EvaGreen®	1_T0	73.70
SsoFast™ EvaGreen®	8_T1	73.70
SsoFast™ EvaGreen®	8_T1	73.70
SsoFast™ EvaGreen®	8_T1	73.70
SsoFast™ EvaGreen®	1_T2	73.90
SsoFast™ EvaGreen®	1_T2	73.70
SsoFast™ EvaGreen®	1_T2	73.70
SsoFast™ EvaGreen®	8_T0	73.70
SsoFast™ EvaGreen®	8_T0	73.70
SsoFast™ EvaGreen®	8_T0	73.70
SsoFast™ EvaGreen®	3_T1	None
SsoFast™ EvaGreen®	3_T1	73.70
SsoFast™ EvaGreen®	3_T1	73.70
SsoFast™ EvaGreen®	8_T2	73.70
SsoFast™ EvaGreen®	8_T2	73.70
SsoFast™ EvaGreen®	8_T2	73.70
SsoFast™ EvaGreen®	3_T0	None
SsoFast™ EvaGreen®	3_T0	73.80
SsoFast™ EvaGreen®	3_T0	73.80
SsoFast™ EvaGreen®	9_T1	73.70
SsoFast™ EvaGreen®	9_T1	73.70
SsoFast™ EvaGreen®	9_T1	73.70
SsoFast™ EvaGreen®	6_T1	73.80
SsoFast™ EvaGreen®	6_T1	73.70
SsoFast™ EvaGreen®	6_T1	73.70
SsoFast™ EvaGreen®	9_T0	73.60
SsoFast™ EvaGreen®	9_T0	73.70
SsoFast™ EvaGreen®	6_T0	73.80
SsoFast™ EvaGreen®	6_T0	73.70
SsoFast™ EvaGreen®	6_T0	73.70
SsoFast™ EvaGreen®	7_T1	None
SsoFast™ EvaGreen®	7_T1	73.60
SsoFast™ EvaGreen®	7_T1	73.70
SsoFast™ EvaGreen®	PC_SC	73.70
SsoFast™ EvaGreen®	PC_SC	73.60
SsoFast™ EvaGreen®	PC_SC	73.70
SsoFast™ EvaGreen®	MB	None
SsoFast™ EvaGreen®	MB	None
SsoFast™ EvaGreen®	MB	None

Sample ID: # Tank, T0 Time zero, T1 Time 1 (24 hrs after removal), T2 Time 2 (48 hours after removal), PC_SC Signal crayfish positive DNA control, MB Amplification negative control.

**Table 4:** Melt peak data for HOT FIREPol^®^ EvaGreen^®^ qPCR multiplex optimised reactions. 164 – 287 = *Aphanomyces astaci*-infected *Pacifastacus leniusculus* individuals.

**Mastermix**	**Sample ID**	**Melt Temperature (°C)**
HOT FIREPol^®^ EvaGreen^®^	287	82.90
HOT FIREPol^®^ EvaGreen^®^	287	75.80
HOT FIREPol^®^ EvaGreen^®^	287	82.90
HOT FIREPol^®^ EvaGreen^®^	287	75.80
HOT FIREPol^®^ EvaGreen^®^	287	82.90
HOT FIREPol^®^ EvaGreen^®^	287	75.80
HOT FIREPol^®^ EvaGreen^®^	281	82.80
HOT FIREPol^®^ EvaGreen^®^	281	75.90
HOT FIREPol^®^ EvaGreen^®^	281	82.70
HOT FIREPol^®^ EvaGreen^®^	281	75.90
HOT FIREPol^®^ EvaGreen^®^	281	82.80
HOT FIREPol^®^ EvaGreen^®^	281	75.90
HOT FIREPol^®^ EvaGreen^®^	164	82.70
HOT FIREPol^®^ EvaGreen^®^	164	75.90
HOT FIREPol^®^ EvaGreen^®^	164	82.30
HOT FIREPol^®^ EvaGreen^®^	164	75.90
HOT FIREPol^®^ EvaGreen^®^	164	82.80
HOT FIREPol^®^ EvaGreen^®^	164	75.80
HOT FIREPol^®^ EvaGreen^®^	278	82.30
HOT FIREPol^®^ EvaGreen^®^	278	75.90
HOT FIREPol^®^ EvaGreen^®^	278	82.80
HOT FIREPol^®^ EvaGreen^®^	278	75.80
HOT FIREPol^®^ EvaGreen^®^	278	82.80
HOT FIREPol^®^ EvaGreen^®^	278	75.80
HOT FIREPol^®^ EvaGreen^®^	MB	None
HOT FIREPol^®^ EvaGreen^®^	MB	None
HOT FIREPol^®^ EvaGreen^®^	MB	None

Sample ID: # Infected crayfish individual.

**Fig. 7:**
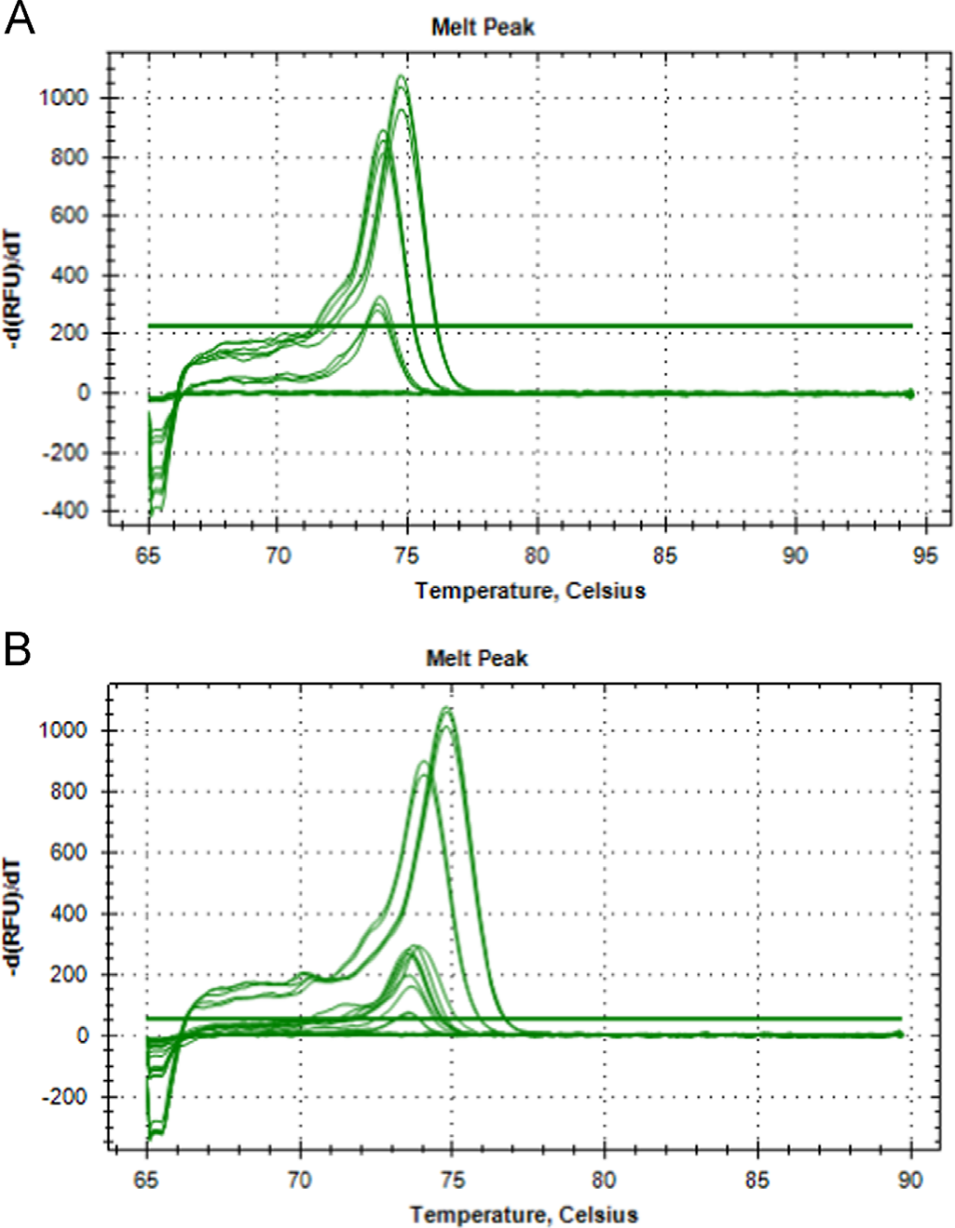
Melt peaks from SsoFast™ EvaGreen® eDNA qPCR amplifications for the trap water samples from the Bachowey (A) and Duhonw catchments (B).

In Section 1.4, data represents SsoFast™ EvaGreen®qPCR product melt curve graphs (Fig. 7) and raw melt output (Table 5) from positive eDNA water sample amplifications collected in the Bachowey and Duhonw rivers around crayfish traps containing *P. leniusculus.*

**Table 5:** Melt peak data from SsoFast™ EvaGreen® eDNA qPCR amplifications for the trap water samples from the Bachowey and Duhonw catchments.

**Mastermix**	**Catchment**	**Sample ID**	**Melt Temperature (°C)**
SsoFast™ EvaGreen®	Bachowey	L3B	73.80
SsoFast™ EvaGreen®	Bachowey	L3B	73.90
SsoFast™ EvaGreen®	Bachowey	L3B	73.90
SsoFast™ EvaGreen®	Bachowey	L4A	73.90
SsoFast™ EvaGreen®	Bachowey	L4A	73.70
SsoFast™ EvaGreen®	Bachowey	L4A	73.70
SsoFast™ EvaGreen®	Bachowey	L4B	73.80
SsoFast™ EvaGreen®	Bachowey	L4B	73.70
SsoFast™ EvaGreen®	Bachowey	L4B	73.70
SsoFast™ EvaGreen®	Duhonw	L5B	73.70
SsoFast™ EvaGreen®	Duhonw	L5B	73.70
SsoFast™ EvaGreen®	Duhonw	L5B	73.70
SsoFast™ EvaGreen®	Duhonw	L5C	None
SsoFast™ EvaGreen®	Duhonw	L5C	None
SsoFast™ EvaGreen®	Duhonw	L5C	None
SsoFast™ EvaGreen®	N/A	PC_SC	74.10
SsoFast™ EvaGreen®	N/A	PC_SC	74.00
SsoFast™ EvaGreen®	N/A	PC_SC	74.00
SsoFast™ EvaGreen®	N/A	PC_NC	74.80
SsoFast™ EvaGreen®	N/A	PC_NC	74.80
SsoFast™ EvaGreen®	N/A	PC_NC	74.80
SsoFast™ EvaGreen®	N/A	MB	None
SsoFast™ EvaGreen®	N/A	MB	None
SsoFast™ EvaGreen®	N/A	MB	None
SsoFast™ EvaGreen®	N/A	MB	None
SsoFast™ EvaGreen®	N/A	MB	None
SsoFast™ EvaGreen®	N/A	MB	None

Sample ID: L# Location number with subsample letter, PC_SC Signal crayfish positive DNA control, PC_NC Native crayfish positive DNA control, MB Amplification negative control.

**Fig. 8:**
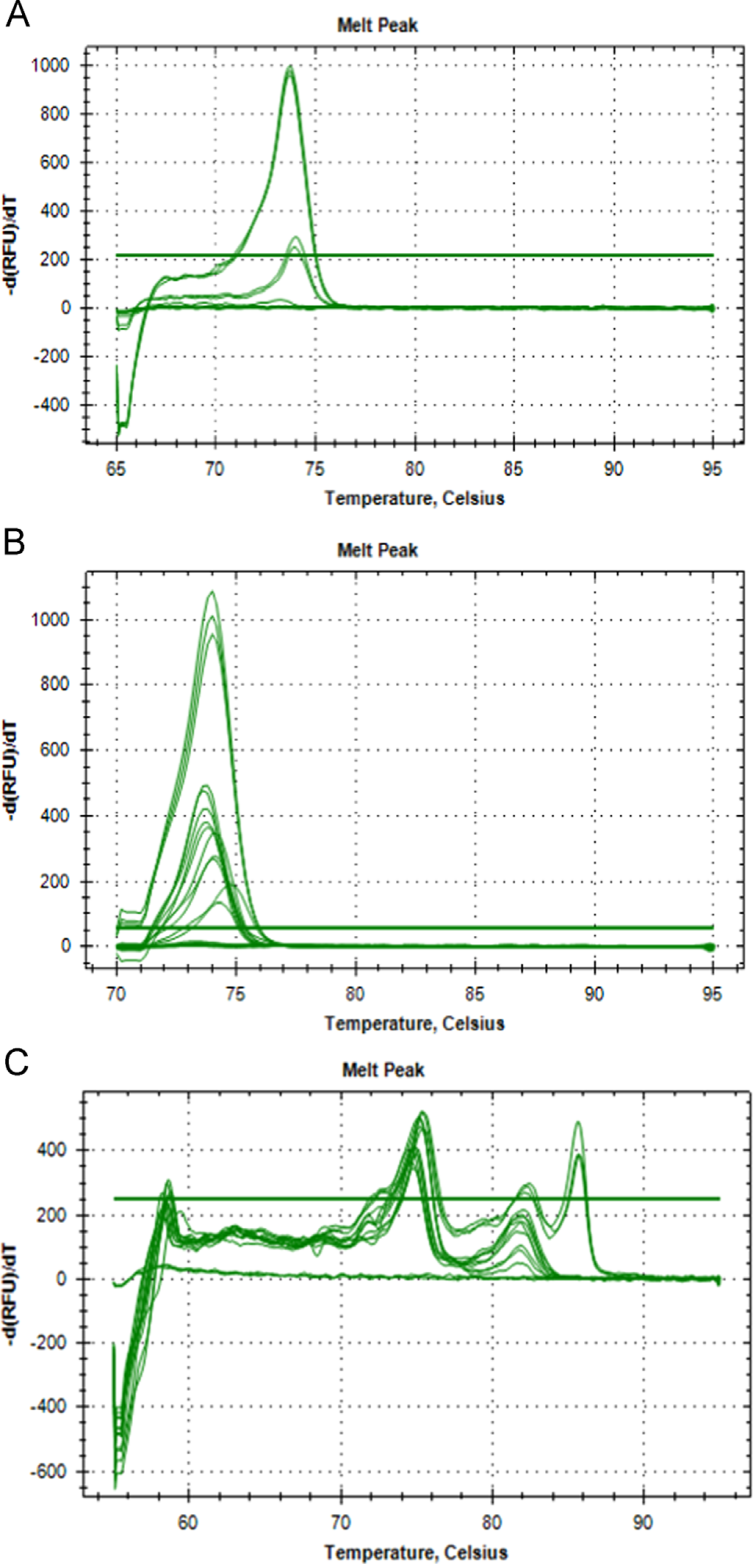
Melt peaks from SsoFast™ EvaGreen® eDNA qPCR amplifications for the Bachowey catchment 2015 samples (A), Sgithwen and Bachowey catchment 2016 samples (B) and HOT FIREPol^®^ EvaGreen^®^ eDNA qPCR amplifications from Bachowey (C).

Section 1.5 contains both qPCR melt curve graphs and raw melt information from positive amplifications from the Sgithwen and Bachowey catchments using both SsoFast™ EvaGreen® and HOT FIREPol® EvaGreen® mastermixes (Fig. 8, Table 6).

**Table 6:** Melt peak data from SsoFast™ EvaGreen® eDNA qPCR amplifications for the Bachowey catchment 2015 samples and Sgithwen and Bachowey catchment 2016 samples.

**Mastermix**	**Catchment**	**Year**	**Sample ID**	**Melt Temperature (°C)**
SsoFast™ EvaGreen®	Sgithwen	2017	2B	74.80
SsoFast™ EvaGreen®	Sgithwen	2017	2B	74.80
SsoFast™ EvaGreen®	Bachowey	2016	3	73.70
SsoFast™ EvaGreen®	Bachowey	2016	3	73.70
SsoFast™ EvaGreen®	Bachowey	2016	3	73.70
SsoFast™ EvaGreen®	Bachowey	2017	4G	74.00
SsoFast™ EvaGreen®	Bachowey	2017	4G	74.20
SsoFast™ EvaGreen®	Bachowey	2017	4G	74.20
SsoFast™ EvaGreen®	Bachowey	2017	4F	73.80
SsoFast™ EvaGreen®	Bachowey	2017	4F	73.80
SsoFast™ EvaGreen®	Bachowey	2017	4I	73.80
SsoFast™ EvaGreen®	Bachowey	2017	4I	73.80
SsoFast™ EvaGreen®	Bachowey	2017	4I	73.70
SsoFast™ EvaGreen®	N/A	N/A	PC_SC	74.00
SsoFast™ EvaGreen®	N/A	N/A	PC_SC	74.00
SsoFast™ EvaGreen®	N/A	N/A	PC_SC	73.90
SsoFast™ EvaGreen®	N/A	N/A	PC_NC	74.80
SsoFast™ EvaGreen®	N/A	N/A	PC_NC	74.80
SsoFast™ EvaGreen®	N/A	N/A	PC_NC	74.80
SsoFast™ EvaGreen®	N/A	N/A	MB	None
SsoFast™ EvaGreen®	N/A	N/A	MB	None
SsoFast™ EvaGreen®	N/A	N/A	MB	None
SsoFast™ EvaGreen®	N/A	N/A	EB1	None
SsoFast™ EvaGreen®	N/A	N/A	EB1	None
SsoFast™ EvaGreen®	N/A	N/A	EB1	None
SsoFast™ EvaGreen®	N/A	N/A	EB2	None
SsoFast™ EvaGreen®	N/A	N/A	EB2	None
SsoFast™ EvaGreen®	N/A	N/A	EB2	None
SsoFast™ EvaGreen®	N/A	N/A	EB3	None
SsoFast™ EvaGreen®	N/A	N/A	EB3	None
SsoFast™ EvaGreen®	N/A	N/A	EB3	None
HOT FIREPol^®^ EvaGreen^®^	Bachowey	2016	10	75.10
HOT FIREPol^®^ EvaGreen^®^	Bachowey	2016	10	83.90
HOT FIREPol^®^ EvaGreen^®^	Bachowey	2016	10	75.20
HOT FIREPol^®^ EvaGreen^®^	Bachowey	2016	10	83.00
HOT FIREPol^®^ EvaGreen^®^	Bachowey	2016	10	75.00
HOT FIREPol^®^ EvaGreen^®^	Bachowey	2016	10	82.90
HOT FIREPol^®^ EvaGreen^®^	Bachowey	2016	11	75.20
HOT FIREPol^®^ EvaGreen^®^	Bachowey	2016	11	82.90
HOT FIREPol^®^ EvaGreen^®^	Bachowey	2016	11	75.20
HOT FIREPol^®^ EvaGreen^®^	Bachowey	2016	11	82.80
HOT FIREPol^®^ EvaGreen^®^	Bachowey	2016	11	75.20
HOT FIREPol^®^ EvaGreen^®^	Bachowey	2016	11	82.90
HOT FIREPol^®^ EvaGreen^®^	Bachowey	2016	14	75.20
HOT FIREPol^®^ EvaGreen^®^	Bachowey	2016	14	83.00
HOT FIREPol^®^ EvaGreen^®^	Bachowey	2016	14	75.20
HOT FIREPol^®^ EvaGreen^®^	Bachowey	2016	14	83.00
HOT FIREPol^®^ EvaGreen^®^	Bachowey	2016	14	75.10
HOT FIREPol^®^ EvaGreen^®^	Bachowey	2016	14	82.90
HOT FIREPol^®^ EvaGreen^®^	Bachowey	2016	14	75.10
HOT FIREPol^®^ EvaGreen^®^	Bachowey	2016	14	82.90
HOT FIREPol^®^ EvaGreen^®^	Bachowey	2016	14	75.60
HOT FIREPol^®^ EvaGreen^®^	Bachowey	2016	14	83.00
HOT FIREPol^®^ EvaGreen^®^	N/A	N/A	PC POOL	75.70
HOT FIREPol^®^ EvaGreen^®^	N/A	N/A	PC POOL	82.90
HOT FIREPol^®^ EvaGreen^®^	N/A	N/A	PC POOL	75.70
HOT FIREPol^®^ EvaGreen^®^	N/A	N/A	PC POOL	83.00
HOT FIREPol^®^ EvaGreen^®^	N/A	N/A	PC POOL	75.70
HOT FIREPol^®^ EvaGreen^®^	N/A	N/A	PC POOL	82.90
HOT FIREPol^®^ EvaGreen^®^	N/A	N/A	MB	None
HOT FIREPol^®^ EvaGreen^®^	N/A	N/A	MB	None
HOT FIREPol^®^ EvaGreen^®^	N/A	N/A	MB	None

Sample ID: # Wye catchment sample with corresponding subsample letter, PC_SC Signal crayfish positive DNA control, PC_NC Native crayfish positive DNA control, MB Amplification negative control, EB# Extraction negative control.

**Fig. 9:**
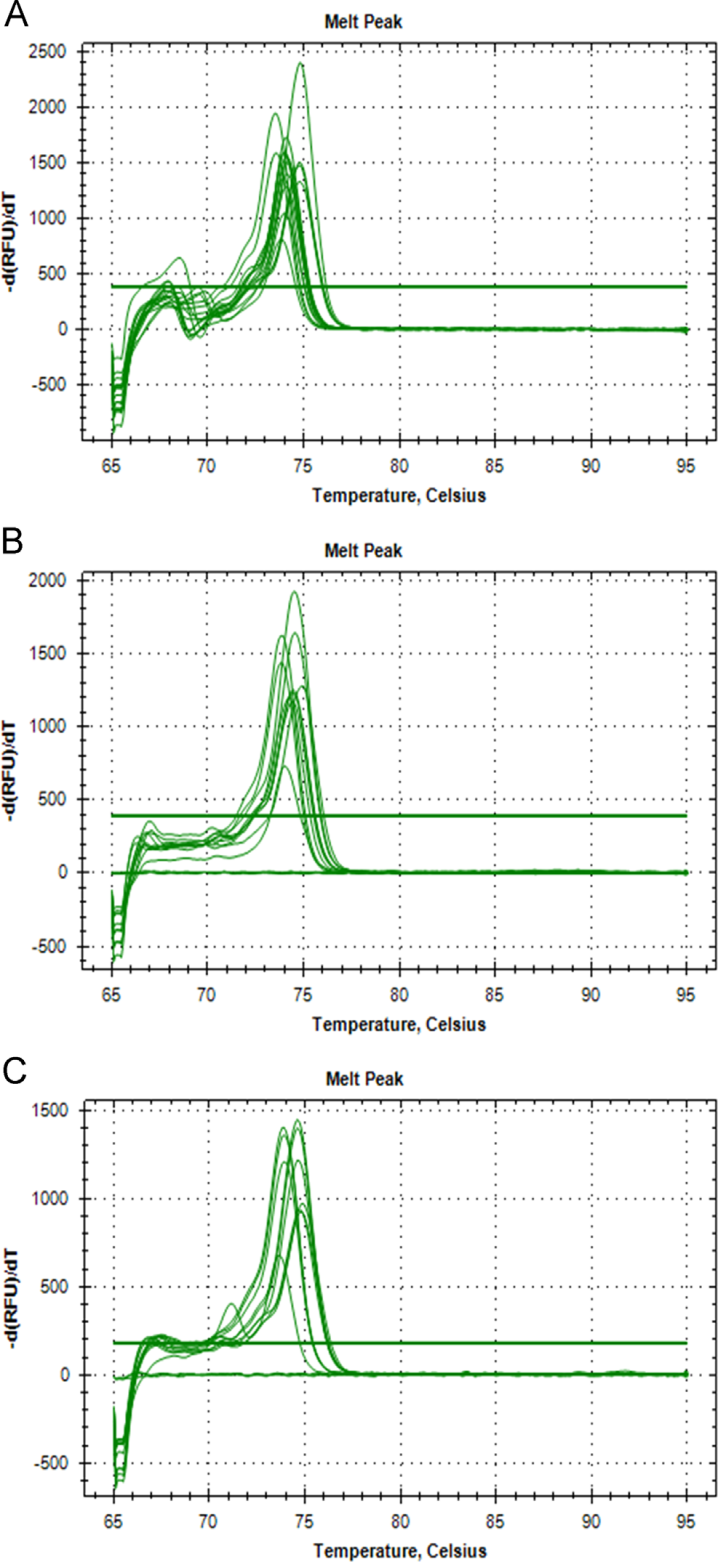
Melt peaks from SsoFast™ EvaGreen® eDNA qPCR amplifications for the 2016 Medway catchment site 5 (A), 2017 Medway catchment site 5 (B) and Itchen catchment at site 8 (C).

Data displayed in Section 1.6 includes the SsoFast™ EvaGreen®qPCR product melt curve graphs and raw melt data from positive detections of both *P. leniusculus* and *A. pallipes* at the same site in the River Medway and Itchen (Fig. 9, Table 7). To conclude, Table 8 provides raw melt data on the absence of *A. astaci* DNA at sites in the River Medway and Itchen where both *P. leniusculus* and *A. pallipes* DNA were detected.

**Table 7:** Melt peak data from SsoFast™ EvaGreen® eDNA qPCR amplifications for the 2016 Medway catchment site 5, 2017 Medway catchment site 5 and Itchen catchment at site 8.

**Mastermix**	**Catchment**	**Year**	**Sample ID**	**Melt Temperature (°C)**
SsoFast™ EvaGreen®	Medway	2016	M5A	68.00
SsoFast™ EvaGreen®	Medway	2016	M5A	74.10
SsoFast™ EvaGreen®	Medway	2016	M5A	74.10
SsoFast™ EvaGreen®	Medway	2016	M5A	None
SsoFast™ EvaGreen®	Medway	2016	M5B	67.90
SsoFast™ EvaGreen®	Medway	2016	M5B	74.00
SsoFast™ EvaGreen®	Medway	2016	M5B	68.00
SsoFast™ EvaGreen®	Medway	2016	M5B	74.00
SsoFast™ EvaGreen®	Medway	2016	M5B	68.50
SsoFast™ EvaGreen®	Medway	2016	M5B	74.80
SsoFast™ EvaGreen®	Medway	2016	M5C	74.10
SsoFast™ EvaGreen®	Medway	2016	M5C	74.10
SsoFast™ EvaGreen®	Medway	2016	M5C	74.80
SsoFast™ EvaGreen®	Medway	2016	M5D	74.10
SsoFast™ EvaGreen®	Medway	2016	M5D	74.00
SsoFast™ EvaGreen®	Medway	2016	M5D	74.80
SsoFast™ EvaGreen®	Medway	2016	M5E	67.90
SsoFast™ EvaGreen®	Medway	2016	M5E	74.00
SsoFast™ EvaGreen®	Medway	2016	M5E	73.80
SsoFast™ EvaGreen®	Medway	2016	M5E	74.80
SsoFast™ EvaGreen®	N/A	N/A	PC_SC	73.70
SsoFast™ EvaGreen®	N/A	N/A	PC_SC	73.70
SsoFast™ EvaGreen®	N/A	N/A	PC_NC	74.80
SsoFast™ EvaGreen®	N/A	N/A	PC_NC	74.80
SsoFast™ EvaGreen®	N/A	N/A	MB	None
SsoFast™ EvaGreen®	N/A	N/A	MB	None
SsoFast™ EvaGreen®	N/A	N/A	MB	None
SsoFast™ EvaGreen®	Medway	2017	M5B	74.00
SsoFast™ EvaGreen®	Medway	2017	M5B	None
SsoFast™ EvaGreen®	Medway	2017	M5B	74.00
SsoFast™ EvaGreen®	Medway	2017	M5C	74.60
SsoFast™ EvaGreen®	Medway	2017	M5C	74.60
SsoFast™ EvaGreen®	Medway	2017	M5C	74.60
SsoFast™ EvaGreen®	N/A	N/A	PC_SC	73.90
SsoFast™ EvaGreen®	N/A	N/A	PC_SC	73.80
SsoFast™ EvaGreen®	N/A	N/A	PC_SC	73.90
SsoFast™ EvaGreen®	N/A	N/A	PC_NC	74.60
SsoFast™ EvaGreen®	N/A	N/A	PC_NC	74.60
SsoFast™ EvaGreen®	N/A	N/A	PC_NC	74.90
SsoFast™ EvaGreen®	N/A	N/A	MB	None
SsoFast™ EvaGreen®	N/A	N/A	MB	None
SsoFast™ EvaGreen®	N/A	N/A	MB	None
SsoFast™ EvaGreen®	Itchen	2017	I8E	74.00
SsoFast™ EvaGreen®	Itchen	2017	I8E	74.00
SsoFast™ EvaGreen®	Itchen	2017	I8F	74.00
SsoFast™ EvaGreen®	Itchen	2017	I8F	74.00
SsoFast™ EvaGreen®	Itchen	2017	I8A	73.70
SsoFast™ EvaGreen®	Itchen	2017	I8A	73.70
SsoFast™ EvaGreen®	Itchen	2017	I8C	74.80
SsoFast™ EvaGreen®	Itchen	2017	I8C	74.90
SsoFast™ EvaGreen®	Itchen	2017	I8C	74.70
SsoFast™ EvaGreen®	N/A	N/A	PC_SC	73.90
SsoFast™ EvaGreen®	N/A	N/A	PC_SC	73.90
SsoFast™ EvaGreen®	N/A	N/A	PC_SC	73.90
SsoFast™ EvaGreen®	N/A	N/A	PC_NC	74.60
SsoFast™ EvaGreen®	N/A	N/A	PC_NC	74.60
SsoFast™ EvaGreen®	N/A	N/A	PC_NC	74.60
SsoFast™ EvaGreen®	N/A	N/A	MB	None
SsoFast™ EvaGreen®	N/A	N/A	MB	None
SsoFast™ EvaGreen®	N/A	N/A	MB	None

Sample ID: M# Medway catchment sample with corresponding subsample letter, I# Itchen catchment sample with corresponding subsample letter, PC_SC Signal crayfish positive DNA control, PC_NC Native crayfish positive DNA control, MB Amplification negative control.

**Table 8:** Melt peak data from HOT FIREPol^®^ EvaGreen^®^ eDNA qPCR amplifications from the Medway and Itchen catchments, at sites where both *Pacifastacus leniusculus* and *Austropotamobius pallipes* DNA was detected in the same site.

**Mastermix**	**Catchment**	**Year**	**Sample ID**	**Melt Temperature (°C)**
HOT FIREPol^®^ EvaGreen^®^	Medway	2016	M5A	76.90
HOT FIREPol^®^ EvaGreen^®^	Medway	2016	M5A	76.00
HOT FIREPol^®^ EvaGreen^®^	Medway	2016	M5A	76.00
HOT FIREPol^®^ EvaGreen^®^	Medway	2016	M5B	76.00
HOT FIREPol^®^ EvaGreen^®^	Medway	2016	M5B	76.00
HOT FIREPol^®^ EvaGreen^®^	Medway	2016	M5B	75.90
HOT FIREPol^®^ EvaGreen^®^	Medway	2016	M5C	None
HOT FIREPol^®^ EvaGreen^®^	Medway	2016	M5C	75.90
HOT FIREPol^®^ EvaGreen^®^	Medway	2016	M5C	75.90
HOT FIREPol^®^ EvaGreen^®^	Medway	2016	M5D	75.90
HOT FIREPol^®^ EvaGreen^®^	Medway	2016	M5D	None
HOT FIREPol^®^ EvaGreen^®^	Medway	2016	M5D	75.90
HOT FIREPol^®^ EvaGreen^®^	Medway	2016	M5E	75.80
HOT FIREPol^®^ EvaGreen^®^	Medway	2016	M5E	None
HOT FIREPol^®^ EvaGreen^®^	Medway	2016	M5E	None
HOT FIREPol^®^ EvaGreen^®^	Medway	2016	M5F	75.90
HOT FIREPol^®^ EvaGreen^®^	Medway	2016	M5F	75.90
HOT FIREPol^®^ EvaGreen^®^	Medway	2016	M5F	75.80
HOT FIREPol^®^ EvaGreen^®^	Medway	2017	M5C	75.90
HOT FIREPol^®^ EvaGreen^®^	Medway	2017	M5C	75.90
HOT FIREPol^®^ EvaGreen^®^	Medway	2017	M5C	76.90
HOT FIREPol^®^ EvaGreen^®^	Itchen	2017	I8C	76.90
HOT FIREPol^®^ EvaGreen^®^	Itchen	2017	I8C	76.90
HOT FIREPol^®^ EvaGreen^®^	Itchen	2017	I8C	None
HOT FIREPol^®^ EvaGreen^®^	Itchen	2017	I8F	75.90
HOT FIREPol^®^ EvaGreen^®^	Itchen	2017	I8F	None
HOT FIREPol^®^ EvaGreen^®^	Itchen	2017	I8F	None
HOT FIREPol^®^ EvaGreen^®^	N/A	N/A	PC_SC	75.90
HOT FIREPol^®^ EvaGreen^®^	N/A	N/A	PC_SC	76.00
HOT FIREPol^®^ EvaGreen^®^	N/A	N/A	PC_SC	75.90
HOT FIREPol^®^ EvaGreen^®^	N/A	N/A	PC_AA	82.90
HOT FIREPol^®^ EvaGreen^®^	N/A	N/A	PC_AA	82.90
HOT FIREPol^®^ EvaGreen^®^	N/A	N/A	PC_AA	82.90
HOT FIREPol^®^ EvaGreen^®^	N/A	N/A	MB	None
HOT FIREPol^®^ EvaGreen^®^	N/A	N/A	MB	None
HOT FIREPol^®^ EvaGreen^®^	N/A	N/A	MB	None

Sample ID: M# Medway catchment sample with corresponding subsample letter, I# Itchen catchment sample with corresponding subsample letter, PC_SC Signal crayfish positive DNA control, PC_NC Native crayfish positive DNA control, PC_AA Crayfish plague positive DNA control, MB Amplification negative control.

### 1.1 Sequence alignment of 16s mtDNA qPCR product for target crayfish species

See Fig. 1.

### 1.2 eDNA yield data from ex-situ samples

See Fig. 2.

### 1.3 Data on qPCR optimisation for SYBR™ Green, SsoFast™ EvaGreen® and HOT FIREPol® EvaGreen® mastermixes

See Fig. 3.

### 1.4 Positive trap water sample amplifications for Pacifastacus leniusculus in the Wye catchment

See Fig. 7.

### 1.5 Data from positive field eDNA amplifications for Pacifastacus leniusculus, Austropotamobius pallipes and Aphanomyces astaci in the Wye catchment

See Fig. 8.

### 1.6 Data from field eDNA samples positive for *Pacifastacus leniusculus* and *Austropotamobius pallipes* in the same site in the River Medway and Itchen

See Fig. 9.

## 2 Experimental design, materials and methods

Methodologies that produced the data presented in this article are fully detailed in [1]. Below, the qPCR protocol for both SsoFast™ EvaGreen® and HOT FIREPol® EvaGreen® are described to complement data provided here.

### 2.1 Sample collection

Water samples were collected at six locations in the River Wye catchment, seven sites in the River Taff catchment, both in Wales, and at 29 sites in the Itchen and Medway rivers, Southern England as detailed in [1]. An ex-situ experiment was also conducted with *P. leniusculus* in three 2 L isolated tanks from where water samples were collected 24 and 48 h after removal of the crayfish [1].

### 2.2 qPCR analysis protocol

DNA from the ex-situ eDNA and tissue samples for *P. leniusculus* and *A. pallipes* were extracted using Qiagen® DNeasy Blood and Tissue Kit (Qiagen, UK). Crayfish specific primers were designed using Primer3, then tested using Beacon Primer Designer (ver. 2.1, PREMIER Biosoft), and finally checked for cross-amplification using NCBI Primer-BLAST [2] and fresh tissue samples as described in [1].

Water samples were amplified in triplicate using optimised SsoFast™ EvaGreen® supermix assay to assess presence of *P. leniusculus* and *A.pallipes* through diagnostic melt peak temperature of resulting qPCR products. Reactions were undertaken in 10 µl volumes using a CFX96 Real-Time PCR detection system (Bio-Rad, UK) consisting of 5 µl SsoFast™ EvaGreen® supermix, 0.25 µl each forward and reverse primer (ApalPlen16S), 3.5 µl ultrapure water and 1 µl DNA. PCR protocol began with 15 min of denaturation at 95 °C, followed by 40 cycles of 95 °C for 10 s and 61.5 °C for 30 s. A melt gradient step was applied to the end of RT-qPCR reactions, ranging from 55 °C to 95 °C in 0.1 °C increments. Once qPCR products were analysed for presence/absence of *P. leniusculus* and *A.pallipes*, qPCR amplifications were repeated for positive sites using 2× HOT FIREPol® EvaGreen® multiplex mix with 0.4 µl of primer mix (5 µM), 6.6 µl of ultrapure water and 1 µl template DNA. Cycling conditions were as follows: activation at 95 °C for 12 min, 40 cycles of 95 °C for 15 s, 61.5 °C for 20 s and 72 °C for 20 s. After the PCR reaction, a melt gradient was applied, which ran from 65 °C to 95 °C by raising 1 °C for 10 s each step. Resulting melt peaks from the multiplex qPCR were then assessed to determine presence/absence of *A. astaci* in *P. leniusculus*/*A.pallipes* positive sites.

The results of the ex situ study indicated that DNA concentration decreased slightly but remained fairly constant across the three time points and was still detectable (melt peak above threshold) at the end of the third time point. DNA quantity was fairly uniform across all tanks, which is to be expected as there was equal biomass of crayfish in each tank, which is known to correlate with the amount of eDNA detected in other aquatic species [3], [4].

Our approach is still subject to factors affecting the sensitivity of the eDNA analyses, such as number and type of samples collected, volume of water sampled, types of waterbody sampled and differences in laboratory techniques [5], [6], [7]. Larger water volumes can increase detectability of eDNA, but there is a trade-off between volume and number of samples, and we have shown that our method can detect infected crayfish even in small volume samples, while allowing to maximize coverage [8], [9], [10].

## References

[1] C.V. Robinson, T.M. Uren Webster, J. Cable, J. James, S. Consuegra Simultaneous detection of invasive signal crayfish, endangered white-clawed crayfish and the crayfish plague using environmental DNA. Biological Conservation 222, 241-252.

[2] Ye J., McGinnis S., Madden T.L. (2006). BLAST: improvements for better sequence analysis. Nucleic Acids Res..

[3] Goldberg C., Sepulveda A., Ray A., Baumgardt J., Waits L. (2013). Environmental DNA as a new method for early detection of New Zealand mudsnails (Potamopyrgus antipodarum). Freshw. Sci..

[4] Thomsen P., Kielgast J., Iversen L.L., Wiuf C., Rasmussen M., Gilbert M.T.P., Orlando L., Willerslev E. (2012). Monitoring endangered freshwater biodiversity using environmental DNA. Mol. Ecol..

[5] Dougherty M., Larson E., Renshaw M., Gantz C., Egan S., Erickson D., Lodge D. (2016). Environmental DNA (eDNA) detects the invasive rustycrayfish Orconectes rusticus at low abundances. J. Appl. Ecol..

[6] Ikeda K., Doi H., Tanaka K., Kawai T., Negishi J. (2016). Using environmental DNA to detect an endangered crayfish Cambaroides japonicus in streams. Conserv. Genet. Resour..

[7] Tréguier A., Paillisson J.-M., Dejean T., Valentini A., Schlaepfer M., Roussel J.-M. (2014). Environmental DNA surveillance for invertebratespecies: advantages and technical limitations todetect invasive crayfish Procambarus clarkii infreshwater ponds. J. Appl. Ecol..

[8] Ficetola G.F., Miaud C., Pompanon F., Taberlet P. (2008). Species detection using environmental DNA from water samples. Biol. Lett..

[9] Goldberg C., Strickler K., Pilliod D. (2015). Moving environmental DNA methods from concept to practice for monitoring aquatic macroorganisms. Biol. Conserv..

[10] Rees H., Maddison B., Middleditch D., Patmore J., Gough K. (2014). The detection of aquatic animal species using environmental DNA – a review of eDNA as a survey tool in ecology. J. Appl. Ecol..

